# Supplementary material for: Generative modeling of single-cell time series with PRESCIENT enables prediction of cell trajectories with interventions
Source: Nat Commun. 2021 May 28;12:3222. doi: 10.1038/s41467-021-23518-w (PMC8163769; doi:10.1038/s41467-021-23518-w)
Supplement: Supplementary file 1 — Supplementary Information [file 41467_2021_23518_MOESM1_ESM.pdf]

Supplemental Information

Generative modeling of single-cell population time-series for inferring cell differentiation landscapes

Grace H.T. Yeo, Sachit D. Saksena, David K. Gifford

1 Supplementary Notes

1.1 Selection of model architecture via performance on held-out time-point recovery

Deep learning enables arbitrary parameterizations of the underlying potential function. We hypothesized that more expressive parameterizations of the potential function might improve modeling capacity of the diffusion process. We hence evaluated models of differing complexity on the task of predicting the marginal distribution at a held-out time point using the Weinreb et al. dataset.

We fit models with four different architectures (fully connected neural network models with 1 layer of 1000 units, 1 layer of 4000 units, 2 layers of 200 units, or 2 layers of 400 units, all using soft-plus as the activation function), and using three different regularization strengths (τ = 1e-3, 1e-6, 1e-9). Architectures were chosen such that the 2 layer models had a corresponding 1 layer model with a roughly equivalent total number of parameters. The smaller models (1 layer of 1000 units, 2 layers of 200 units) had 50k parameters and were trained using Adam with a learning rate of 0.01. The larger models (1 layer of 4000 units, 2 layers of 400 units) had 200k parameters and were trained with a learning rate of 0.005, as these models sometimes diverged when trained with a learning rate of 0.01 (Fig. S1b). Models were then fit as described in Methods.

In general, models performed well, outperforming the baselines described except when the regularization strength was too high (i.e. excepting when τ = 1e-3). For both models trained with or without taking into account cell proliferation, we observed that although 1-layer models may perform as well as 2-layer models on training, 2-layer models achieve lower testing distance even when the number of parameters in the 1-layer models was greater than or equivalent to in the 2-layer models (Fig. S1c,e). This could suggest that the 2-layer models are either better able to approximate the potential function, or are easier to fit. We also observed that model training and testing distance was best with a moderate regularization strength of 1e-6 across model architectures (Fig. S1d,e). Models accounting for cell proliferation performed slightly worse at predicting the held-out time point than models that did not account for cell proliferation.

We reasoned that hyper-parameters selected on this task would be transferable to models for fate prediction because good recovery of the held-out time point should imply that the model had been able to find a good approximation of the underlying potential function. We hence use 2-layer models with 400 units and a regularization strength of 1e-6 for subsequent tasks (Fig. S1a). Models with this architecture achieved both the lowest training and testing distance in our experiments.

Since 2-layer models with τ = 1e-6 generally performed well on the Weinreb et al. dataset, we chose to fit models with 2 layers of either 200 units or 400 units on the Veres et al. dataset. As we observed in the experiments for the Weinreb et al. dataset, the 2 layer 400 unit model achieved a lower training distance (Fig. S3c-d). Hence, we also used a 2 layer 400 unit model for the Veres et al. experiments. Further experiments would be needed to study if larger models could lead to overfitting (such that lower training distance does not necessarily imply lower testing distance).

2 Supplementary Figures

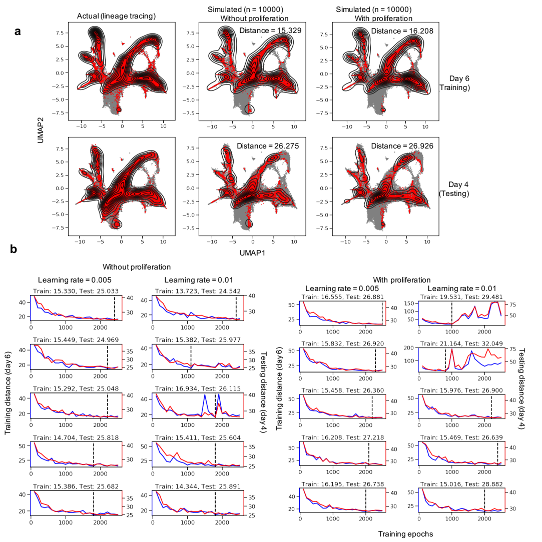

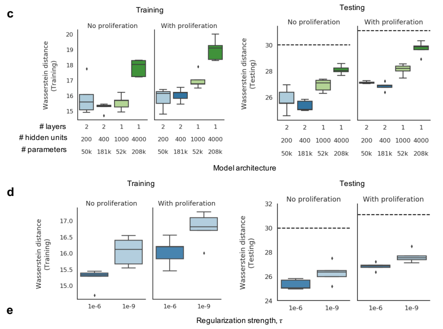

e

Model	No proliferation 1e-3	No proliferation 1e-6	No proliferation 1e-9	With proliferation 1e-3	With proliferation 1e-6	With proliferation 1e-9
**Training (Day 6)**						
1 × 1000	60.53 ± 0.94	15.57 ± 0.48	16.61 ± 0.76	63.61 ± 0.43	16.99 ± 0.54	17.51 ± 0.74
1 × 4000	68.62 ± 1.49	17.82 ± 0.55	18.82 ± 0.59	71.45 ± 0.61	19.00 ± 0.71	19.90 ± 0.51
2 × 200	31.66 ± 9.18	**15.88 ± 1.14**	16.33 ± 0.63	29.91 ± 6.71	**15.82 ± 0.67**	17.17 ± 0.52
2 × 400	25.33 ± 3.21	**15.23 ± 0.30**	16.06 ± 0.44	27.45 ± 6.41	**16.05 ± 0.42**	16.78 ± 0.49
**Testing (Day 4)**						
1 × 1000	49.76 ± 0.23	26.91 ± 0.48	28.50 ± 0.73	53.56 ± 0.90	28.11 ± 0.42	29.41 ± 0.44
1 × 4000	50.60 ± 0.60	28.09 ± 0.33	29.46 ± 0.24	54.19 ± 0.41	29.73 ± 0.52	31.27 ± 0.32
2 × 200	80.13 ± 8.99	**25.78 ± 0.91**	27.40 ± 1.03	89.11 ± 11.20	**27.11 ± 0.09**	28.23 ± 0.94
2 × 400	89.71 ± 9.35	**25.31 ± 0.41**	26.30 ± 0.83	117.31 ± 27.92	**26.82 ± 0.31**	27.64 ± 0.51

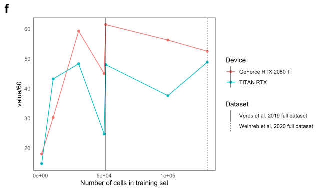

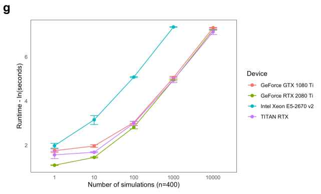

Figure S1: **Training and testing performance on time point recovery on Weinreb et al. lineage tracing dataset.** (a) Results for for 2-layer models with 400 units and a regularization strength of $\tau = 1e{-}6$. Actual and simulated populations on days 6 (training, top), and 4 (testing, below). Distance reported is the Wasserstein distance of the simulated population to the actual population at the given time point. (b) Training (blue, left axis) and testing (red, right axis) performance across training epochs for 2 layer 400 unit models with and without proliferation and with different learning rates across 5 seeds. Training and testing performance is reported for best training epoch, as indicated by the vertical dashed line. (c)-(e) Testing performance for models of different model architecture (c,e) and regularization strength (d,e) for 5 seeds. (c,d) Dashed line indicates linear interpolation baseline. (f) Runtime estimates of training 500 pre-training epochs and 2500 training epochs with fixed hyperparameters on different GPUs (1x) with increasing training set sizes. (g) Runtime estimates of increasing numbers of simulations of 400 randomly initialized cells. Repeated 5 times for each number of simulations and GPU device (1x).

In (c-d), boxplots indicate median (middle line), first and third quartiles (box), and the upper whisker extends from the edges to the largest value no further than 1.5IQR (interquartile range) from the quartiles and the lower whisker extends from the edge to the smallest value at most 1.5IQR of the edge, while data beyond the end of the whiskers are outlying points that are plotted individually as diamonds.

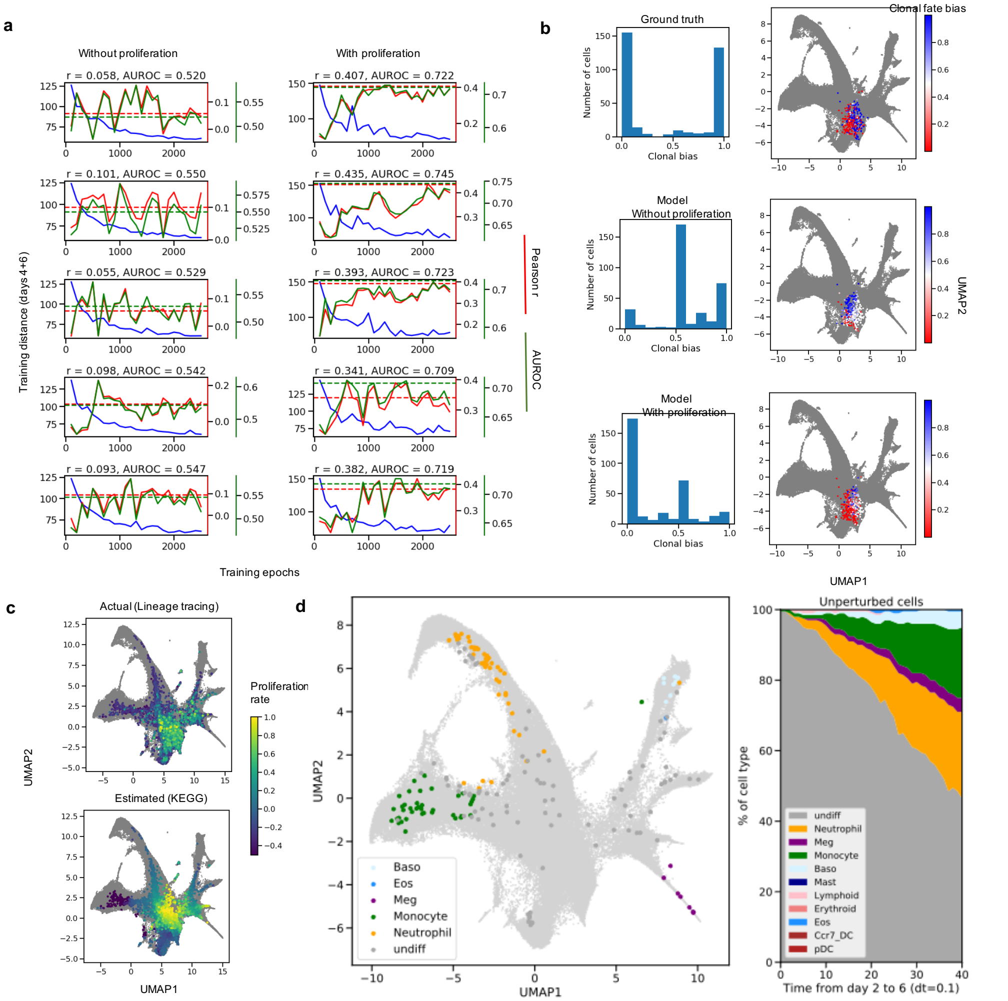

Figure S2: **Training and testing performance on cell fate prediction on Weinreb et al. lineage tracing dataset** (a) Training (blue, left axis) and testing Pearson r (red, first right axis) and AUROC (green, second right axis) across training epochs for 2-layer 400 unit models with and without proliferation. Ensembled testing performance is reported and indicated as horizontal dashed lines (b) Distribution of ground truth and predicted clonal fate bias across testing cells as a histogram (left) and visualized on the UMAP (right) (c) Visualization of actual and estimated proliferation on UMAP (d-e) Distribution of cell types at the final time point (d) and across training steps until final time point (e) for unperturbed simulations of in vitro hematopoietic differentiation.

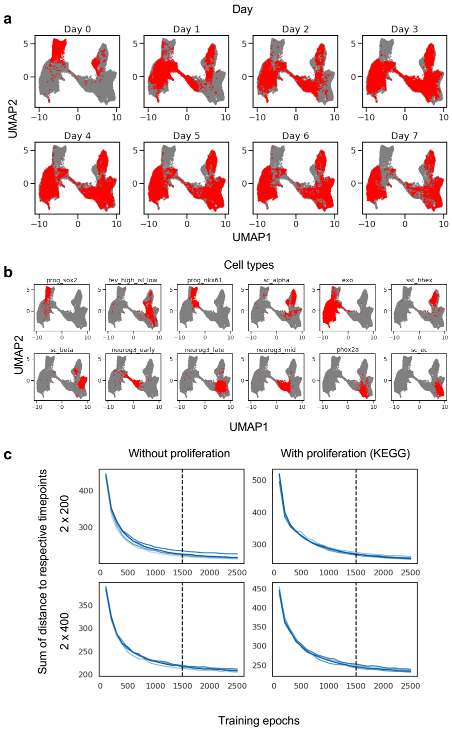

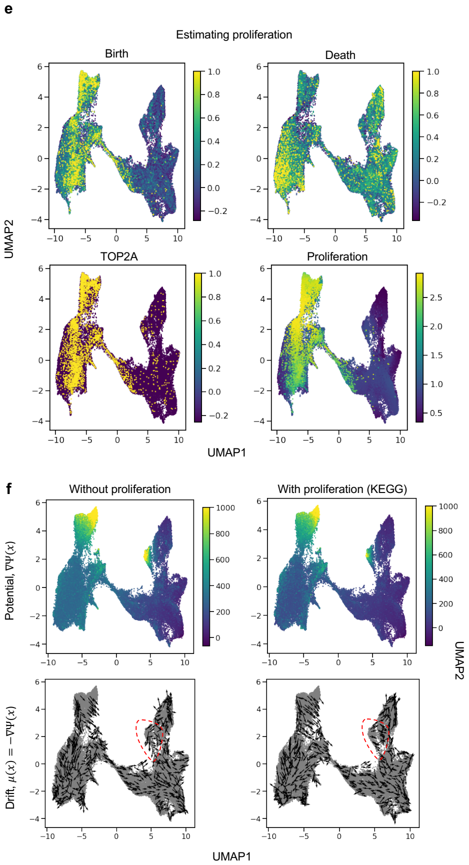

d

Time point	No proliferation 2 × 200	No proliferation 2 × 400	With proliferation (KEGG) 2 × 200	With proliferation (KEGG) 2 × 400
1	37.27 ± 0.78	35.19 ± 0.53	47.70 ± 1.28	44.51 ± 0.98
2	36.12 ± 0.68	34.45 ± 0.69	45.53 ± 1.28	41.04 ± 1.20
3	27.82 ± 0.65	26.87 ± 0.57	33.01 ± 0.65	30.66 ± 0.45
4	30.46 ± 1.12	28.63 ± 0.73	37.39 ± 0.69	33.46 ± 0.65
5	30.20 ± 1.08	28.46 ± 0.44	35.57 ± 0.76	31.90 ± 0.56
6	30.83 ± 1.10	30.17 ± 0.23	34.06 ± 0.48	31.40 ± 0.44
7	32.93 ± 0.77	32.27 ± 0.37	36.60 ± 0.45	33.81 ± 0.61

Figure S3: **Preprocessing and model fitting on Veres et al. dataset.** Visualization of cells at different time points (a) or annotated as different cell types (b) (c-d) Training performance across 5 seed with and without proliferation for 2 2-layer models across training epochs and summed over time points (c) and for individual time points at epoch 1500 (d). The vertical dashed line in (c) also indicates epoch 1500. (e) Visualization of birth, death and proliferation rates estimated via KEGG, as well as scaled expression of TOP2A (f) Visualization of drift and potential learned by example 2 layer, 400 unit evaluated at epoch 1500. The red circles indicate qualitative differences in drift and potential between models fit with and without proliferation.

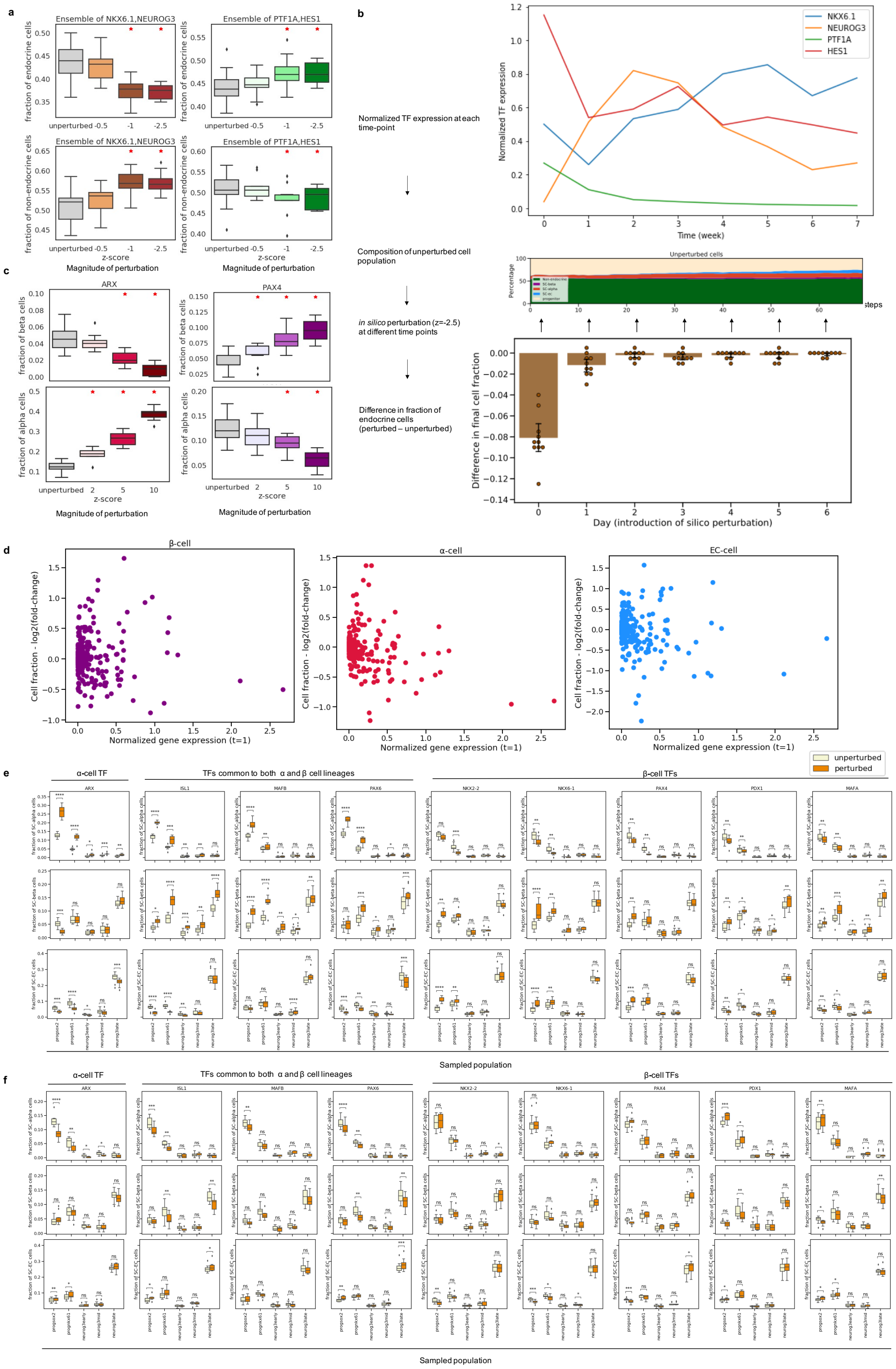

Figure S4: **Expanded evaluation of PRESCIENT perturbational outcomes on Veres et al. 2019 dataset.** (a) Final fractions of endocrine and exocrine cells as a result of ensembled knockdowns of endocrine- and exocrine- associated TFs (left, right) on day 0 (b) *in silico* perturbations (NKX6.1, NEUROG3; z=10) are introduced at different time points to the corresponding unperturbed population. The different outcomes of the cell type of interest are then calculated as the difference in fraction at the final time point starting from the perturbed and unperturbed populations. (c) Final fractions of and cells when introducing perturbations to ARX and PAX4 in the starting cell population at day 0. (a-b) Red asterisks indicate significance at two-sided independent t-test $p < 0.05$ (d) Normalized gene expression at timepoint 0 vs. Log2(fold-change) in cell fractions from perturbations of 200+ TFs (z=5) for β-cells (purple), α-cells (red) and EC-cells (blue). (e-f) Final fractions of α, β and EC cells when starting from different perturbed (e: $z = 5$, f: $z = 5$) vs. unperturbed populations Asterisks indicate significance at paired t-test p * < 0.05 ** < 0.01, *** < 0.001. Different starting populations correspond to cell stages as labeled by Veres et al.: SOX2+ progenitors (progsox2), NKX61+ progenitors (prognkx61), and cells with early/middle/late NEUROG3 signatures (neurog3early, neurog3mid, neurog3late, respectively). (a-c, d-e) Results are reported over 10 randomly sampled starting populations. In b, bar plots show the average fraction of cells with error bars representing the 95% CI. In (a-b), (e-f) boxplots indicate median (middle line), first and third quartiles (box), and the upper whisker extends from the edges to the largest value no further than 1.5IQR (interquartile range) from the quartiles and the lower whisker extends from the edge to the smallest value at most 1.5IQR of the edge, while data beyond the end of the whiskers are outlying points that are plotted individually as diamonds.

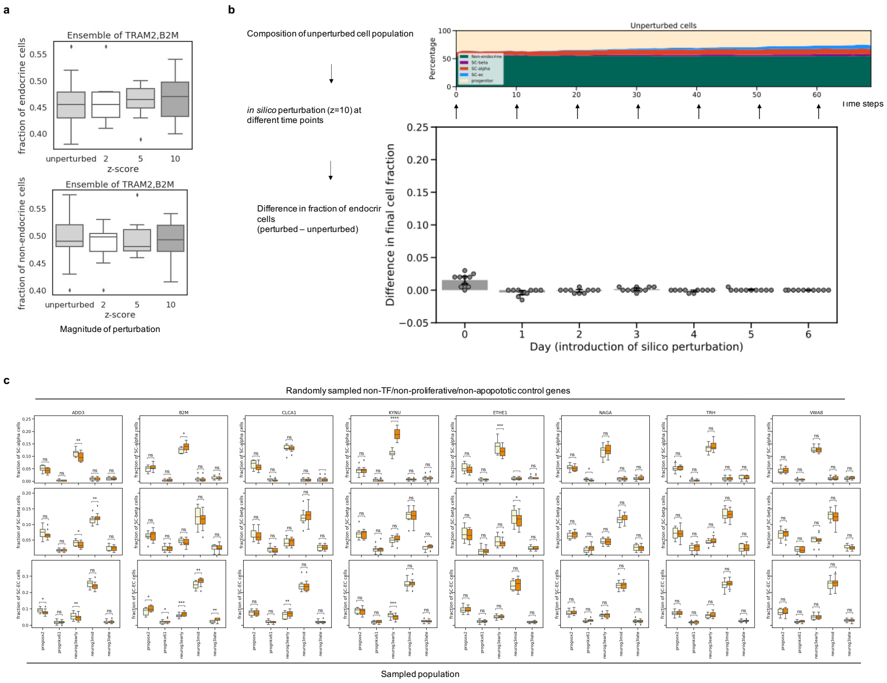

Figure S5: **PRESCIENT predicts non-significant changes to final cell fraction in response to *in silico* perturbations of the non-TFs not involved in apoptosis/proliferation.** (a) Final fractions of endocrine and exocrine cells as a result of ensembled perturbations of non-TF control genes (top, bottom) on day 0. (b) in silico perturbations of non-proliferative, non-TF genes (TRAM2, B2M; z=10) are introduced at different time points to the corresponding unperturbed population. The different outcomes of the cell type of interest are then calculated as the difference in fraction at the final time point starting from the perturbed and unperturbed populations. (c) Final fractions of α, β and EC cells when starting from different perturbed vs. unperturbed populations. Asterisks indicate significance at paired t-test p * < 0.05 ** < 0.01, *** < 0.001. (a-c) Results are reported over 10 randomly sampled starting populations. In b, bar plots show the average fraction of cells with error bars representing the 95% CI. In (a), (c) boxplots indicate median (middle line), first and third quartiles (box), and the upper whisker extends from the edges to the largest value no further than 1.5IQR (interquartile range) from the quartiles and the lower whisker extends from the edge to the smallest value at most 1.5IQR of the edge, while data beyond the end of the whiskers are outlying points that are plotted individually as diamonds.
